# Evaluating the prevalence and adequate treatment for congenital syphilis in a Brazilian reference maternity hospital

**DOI:** 10.1590/1984-0462/2025/43/2024285

**Published:** 2025-08-18

**Authors:** Alan Oliveira Duarte, Mariellen Santos de Jesus Souza, Ricardo Sampaio Hein da Silva, Géssica Almeida Vasconcelos, Lorena Cunha Martins, Maria Aline Silva Alves, Ciro Gomes Machado, Soraia Machado Cordeiro, Carina Carvalho dos Santos, Junia Raquel Dutra Ferreira, Patrícia Santos de Oliveira, Juan Ignacio Calcagno, Isadora Cristina de Siqueira

**Affiliations:** a Fundação Oswaldo Cruz Bahia, Instituto Gonçalo Moniz, Laboratório de Investigação em Saúde Global e Doenças Negligenciadas, Salvador, BA, Brasil.; b Universidade Federal da Bahia, Faculdade de Farmácia, Departamento de Análises Clínicas e Toxicológicas, Salvador, BA, Brasil.; c Secretaria de Saúde do Estado da Bahia, Maternidade Professor José Maria de Magalhães Netto, Salvador, BA, Brasil.

**Keywords:** Congenital syphilis, Newborn health, Public health surveillance, Vertical infection transmission, Prenatal care, Sífilis congênita, Saúde do neonato, Vigilância em saúde pública, Transmissão vertical de infecção, Cuidado pré-natal

## Abstract

**Objective:**

To delineate characteristics associated with congenital syphilis in newborns from a capital city of Brazil, as well as to report prevalence rates, diagnostic approaches, and treatment data through document analysis.

**Methods:**

A cross-sectional, retrospective, and documentary observational study was conducted at a maternity hospital based on congenital syphilis cases reported through notification forms produced between 2016–2019. Statistical analysis involved absolute and relative frequency evaluations.

**Results:**

A total of 879 cases were included, with 2018 being the year with the most cases. Analysis revealed a high proportion of mothers with secondary education (289; 33.0%), and most selfidentifying as mixed-race (400; 45.6%). Infection diagnosis occurred at the time of or following delivery in a large percentage of the women (337; 38.4%). Among newborns, 249 cases (28.3%) exhibited symptoms, with jaundice being the most prevalent (235; 26.9%). While most newborns were discharged following treatment, four cases (0.4%) resulted in syphilis-related fatalities, including one abortion and one stillbirth.

**Conclusions:**

This study highlights deficiencies in the diagnosis and treatment of mothers affected by syphilis, emphasizing the importance of access to early diagnostic testing in pregnant women, as well as newborn screening. We further call attention to the critical need for expanded health education initiatives targeting congenital syphilis awareness among pregnant women and their partners.

## RESUMO

**Objetivo:**

Delinear características associadas à sífilis congênita em recém-nascidos de uma capital do Brasil, bem como relatar taxas de prevalência, abordagens diagnósticas e dados de tratamento por meio de análise documental.

**Métodos:**

Estudo de corte transversal observacional, retrospectivo e documental foi conduzido em uma maternidade, com base nos casos de sífilis congênita reportados por meio de fichas de notificação produzidas entre 2016–2019. A análise estatística envolveu avaliações de frequência absoluta e relativa.

**Resultados:**

Um total de 879 casos foram incluídos, sendo 2018 o ano com mais casos. A análise revelou uma alta proporção de mães com ensino médio (289; 33,0%) e a maioria se autoidentificou como pardas (400; 45,6%). O diagnóstico da infecção ocorreu no momento ou após o parto em uma grande porcentagem das mulheres (337; 38,4%). Entre os recém-nascidos, 249 casos (28,3%) apresentaram sintomas, sendo a icterícia o mais prevalente (235; 26,9%). Enquanto a maioria dos recém-nascidos recebeu alta após o tratamento, quatro casos (0,4%) resultaram em fatalidades relacionadas à sífilis, incluindo um aborto e um natimorto.

**Conclusões:**

Este estudo destaca deficiências no diagnóstico e tratamento de mães afetadas pela sífilis, enfatizando a importância do acesso a testes de diagnóstico precoce em mulheres grávidas, bem como triagem neonatal. Chamamos ainda a atenção para a necessidade crítica de expandir as iniciativas de educação em saúde visando a conscientização sobre a sífilis congênita entre mulheres grávidas e seus parceiros.

## INTRODUCTION

 Congenital syphilis (CS) occurs when *Treponema pallidum*, the causative agent of syphilis, is transmitted vertically from an untreated or inadequately treated pregnant woman to their child. Early diagnosis and prompt treatment are crucial to prevent infection. If unidentified or untreated during pregnancy, this infection can cause a variety of adverse birth outcomes, including miscarriage, stillbirth, neonatal death, preterm birth, low-birthweight, and congenital defects, as well as permanent bone, auditory, and ocular sequelae during child development.^1^

 Serological laboratory techniques are instrumental in CS diagnosis. The interpretation of laboratory results must consider several factors, such as maternal antibody transfer, the serological status of both mother and child, the effectiveness of treatment, and other pertinent laboratory findings. Within this context, the semi-quantitative results obtained from non-treponemal testing of both mothers and their children can aid in diagnosing CS.^2^

 In Brazil, CS is associated with significant neonatal morbidity and mortality. In recent years, the country encompassed 85.0% of CS cases in the Americas.^1,3^ High prevalences of syphilis in pregnancy (SiP) and CS have been observed in many Brazilian states, including the Northeastern state of Bahia, reflecting suboptimal maternal and child health. At a primary public reference maternity hospital in the state capital, Salvador, a previous investigation identified SiP as the most notified infectious disease.^4^

 Previous ecological studies have analyzed Brazil’s CS epidemiological data based on public health system records; however, there is a paucity of studies detailing clinical and laboratory findings of affected newborns. This gap is particularly relevant in regions with high CS prevalence, where a more detailed understanding of newborn presentations can inform targeted interventions. This retrospective observational study was conducted at a reference maternity hospital in Salvador, Bahia, to characterize the epidemiological, sociodemographic, clinical, and laboratory profiles of mothers and newborns diagnosed with CS. By providing local data, this study aimed to enhance knowledge of CS presentation in this high burden setting. Our objective was to delineate the characteristics associated with CS in newborns in this community, describe diagnostic approaches and treatment strategies, and contribute to public health efforts to mitigate the impact of CS in similar contexts. 

## METHOD

 A retrospective, descriptive, documentary study was conducted at the Professor José Maria de Magalhães Netto public reference maternity hospital in 2020. The study included all inborn cases of CS reported between January 2016 and December 2019 at the hospital, based on notification criteria established by the Ministry of Health.^5^ Duplicate notifications or records with missing/invalid information were excluded from the analysis. 

 The Brazilian Ministry of Health established, through its Clinical Protocol and Therapeutic Guidelines for Comprehensive Care for People with Sexually Transmitted Infections,^5^ three scenarios that demand compulsory notification of cases, such as CS, in the National Notifiable Diseases Information System (SINAN). 

 Scenario 1 considers live newborn, stillbirth, or abortion from a pregnant woman who has untreated or inadequately treated SiP. The protocol outlines adequate treatment as the therapeutic regimen consisting of benzathine benzylpenicillin administration, with treatment initiating 30 days before delivery and concluding before delivery, following a therapeutic scheme according to the clinical stage of the infection, and respects the recommended interval between doses. 

 Scenario 2 includes cases of CS-consistent clinical manifestation, a serum quantitative non-treponemal serologic titer that is twofold (or greater) higher than the mother’s titer at delivery, cases that present an increase in non-treponemal serologic titers, or a persistently positive serum non-treponemal test over six months. 

 Scenario 3 comprises microbiological evidence of *T. pallidum* infection in a sample of nasal secretion or skin lesion, biopsy or necropsy of a child, abortion or stillbirth. 

 Sociodemographic, epidemiological, clinical, and laboratory data were extracted from the compulsory notification forms generated by the maternity hospital’s epidemiological surveillance program. Every attempt was made to complement any information missing from the forms using data retrieved from electronic medical records. The medical records were consulted exclusively to complete the missing data in notification forms. To ensure accuracy and security, data were managed using REDCap© 9.3.1 (Vanderbilt University)—a secure web-based software platform specifically designed to facilitate data collection for research studies.^6^ Descriptive statistics were employed, utilizing measures such as absolute and relative frequencies, percentages, means, medians, and interquartile range (IQR) from 25th to 75th percentiles. 

 The present study was approved by the Institutional Review Board of the Faculty of Pharmacy of the Federal University of Bahia (protocol No. 98626718.9.0000.8035). Given the nature of the documentary analysis, which ensured anonymity, the requirement for informed consent forms was waived. 

## RESULTS

**Figure 1:**
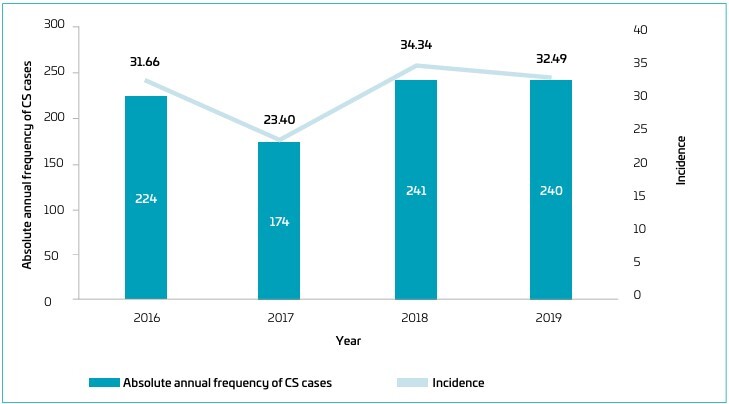
Absolute annual frequency of congenital syphilis (CS) cases and congenital syphilis incidence per 1,000 live births at a reference maternity hospital, from 2016 to 2019 in Salvador (BA), Brazil.

 The maternity hospital reported 887 inborn cases of CS between January 2016 and December 2019. After removing duplicate notifications and excluding records with missing or invalid data, eight cases were omitted, resulting in a final sample of 879 CS cases. During the study period, the annual number of live births at the hospital was 7,075 in 2016, 7,436 in 2017, 7,018 in 2018, and 7,386 in 2019, totaling 28,915 live births. The absolute frequency of CS cases and the hospital’s CS incidence per 1,000 live births for each year are presented in Figure 1. 

 The sociodemographic, clinical, and laboratory profiles of the CS mothers are described in Table 1 and 2. Most mothers were young adults, with a median age of 24 years, who self-identified as mixed-race or black (636; 72.4%). Notably, a significant proportion of mothers completed either full or partial secondary education (289; 32.9%). Regarding prenatal care and the timing of SiP diagnosis, a considerable number of expectant mothers received their SiP diagnosis either at the onset of labor or postpartum (337; 38.4%), even though a high number of mothers reported receiving prenatal care (743; 84.5%). 

**Table 1:** Sociodemographic and clinical characteristics of 879 mothers diagnosed with syphilis at a reference maternity hospital, from 2016 to 2019 in Salvador (BA), Brazil.

Characteristics		n (%)
	Median age, years (IQR)*	24 (20–29)
Area of residence		
	Urban	852 (96.8)
	Metropolitan	8 (0.9)
	Other	6 (0.6)
Skin color (self-reported)		
	Mixed-race	400 (45.6)
	Black	236 (26.8)
	White	24 (2.7)
	Other	8 (0.9)
	Unknown	211 (24.0)
Education level		
	No formal education	3 (0.3)
	Primary	323 (36.8)
	Secondary	289 (32.9)
	University	20 (2.3)
	Unknown	244 (27.7)
Prenatal care		
	Yes	743 (84.5)
	No	84 (9.6)
	Unknown	52 (6.0)
Maternal treatment outcome		
	Adequate	340 (38.7)
	Inadequate	298 (33.9)
	Not performed	137 (15.6)
	Unknown	104 (11.8)
Partner treatment		
	Performed	171 (19.4)
	Not performed	345 (39.2)
	Unknown	363 (41.4)

* IQR: interquartile range (25–75th).

**Table 2:** Diagnosis data of 879 mothers with syphilis at a reference maternity hospital in Salvador (BA), Brazil.

Characteristics		n (%)
Time of syphilis diagnosis		
	During prenatal care	542 (61.6)
	At time of labor	295 (33.6)
	After birth	42 (4.8)
Non-treponemal tests results at delivery		
	Reactive	843 (95.9)
	Non-reactive	26 (3.0)
	Not performed	4 (0.5)
	Unknown	6 (0.7)
Treponemal test results at delivery		
	Reactive	570 (64.9)
	Non-reactive	17 (1.9)
	Not performed	177 (20.1)
	Unknown	115 (13.1)

 The laboratory data extracted from notification forms and patient records revealed that most mothers (863; 98.2%) presented positive serology for *T. pallidum* at the time of labor or curettage. However, the data also highlighted a notable disparity in treponemal testing rates compared to non-treponemal testing; a higher proportion of mothers were not submitted to treponemal testing at the time of labor or curettage (177; 20.2%) compared to non-treponemal testing (4; 0.5%). Furthermore, concerning treatment, the data revealed elevated frequencies of inadequately treated mothers and their sexual partners (33.9% and 39.4%, respectively), signaling a breakdown in the continuum of care for pregnant women afflicted with SiP. Unfortunately, the mothers’ antibiotic regimen was not further detailed in the CS notification forms. 

 The gender distribution of newborns was similar, with 438 male and 435 female cases. Similarly, the racial distribution mirrored that of the mothers, with most newborns reporting as mixed-race or black (449; 51.0%). Table 3 summarizes the demographic and clinical data of the study newborns. 

**Table 3:** Neonatal and clinical characteristics of 879 newborns with congenital syphilis at a reference maternity hospital, from 2016 to 2019 in Salvador (BA), Brazil.

Characteristics		n (%)
	Median age, years (IQR)*	4 (2–11)
Newborn sex		
	Male	438 (49.8)
	Female	435 (49.5)
	Unknown	6 (0.7)
Skin color		
	Mixed-race	305 (34.7)
	Black	144 (16.4)
	White	27 (3.1)
	Others	4 (0.4)
	Unknown	400 (45.4)
Clinical diagnosis		
	Asymptomatic	607 (69.1)
	Symptomatic	249 (28.3)
	Unknown	23 (2.6)
Symptoms		
	Jaundice	235 (26.9)
	Anemia	10 (1.1)
	Hepatomegaly	8 (0.9)
	Skin lesions	7 (0.8)
	Splenomegaly	5 (0.6)
	Osteochondritis	2 (0.2)
	Pseudoparalysis	2 (0.2)
	Muco-bloody rhinitis	1 (0.1)
	Other symptoms	17 (2.0)
Newborn antibiotic therapy		
	Performed	871 (99.1)
	Not performed	7 (0.8)
	Unknown	1 (0.1)
Antibiotic regimen		
	Crystalline penicillin G (100,000 to 150,000 IU/kg/day, for 10 days)	669 (76.2)
	Procaine penicillin G (50,000 IU/kg/day, for 10 days)	116 (13.2)
	Benzathine penicillin G (50,000 IU/kg/day, for one day)	57 (6.5)
	Other penicillin regimen	16 (1.8)
	Other antibiotics	13 (1.5)
Clinical outcome		
	Hospital discharge	866 (98.2)
	Death by other causes	9 (1.1)
	Death by congenital syphilis	2 (0.3)
	Abortion	1 (0.1)
	Stillbirth	1 (0.1)

* IQR: interquartile range (25–75th).

 Most newborns (849; 96.6%) had a positive non-treponemal test performed in peripheral blood. Twenty (4.0%) newborns submitted to lumbar puncture had a positive non-treponemal test in CSF. No treponemal testing was performed at the time of birth, as the Brazilian Ministry of Health dictates. Its guidelines stipulate that such tests should only be administered in children over 18 months of age or older.^5^ Table 4 presents the laboratory findings of the newborns. 

**Table 4:** Laboratory findings of 879 newborns with congenital syphilis at a reference maternity hospital, from 2016 to 2019 in Salvador (BA), Brazil.

Characteristics		n (%)
Non-treponemal test from blood		
	Reactive	849 (96.6)
	Non-reactive	30 (3.4)
Serum VDRL titers at birth		
	1:1	135 (15.9)
	1:2	230 (27.1)
	1:4	155 (18.3)
	1:8	156 (18.4)
	1:16	82 (9.6)
	1:32	41 (4.8)
	1:64	27 (3.2)
	1:128 or higher	15 (1.8)
	Unknown	8 (0.9)
Non-treponemal test of CSF		
	Reactive	20 (4.3*)
	Non-reactive	445 (95.7*)
	Not performed	157 (17.8)
	Unknown	258 (29.3)
Increase in VDRL titer		
	Yes	3 (2.6*)
	No	112 (97.4*)
	Not performed	269 (30.6)
	Unknown	495 (56.4)
Direct evidence of *T. pallidum*		
	Yes	20 (11.8*)
	No	150 (88.2*)
	Not performed	229 (26.0)
	Unknown	480 (54.6)
Abnormal findings on radiography		
	Yes	16 (4*)
	No	389 (96*)
	Not performed	103 (11.7)
	Unknown	372 (42.2)

* percentages were calculated in relation to the number of tests performed.

VDRL: venereal disease research laboratory test (screening for syphilis).

 Among the total 879 newborns, 249 (28.3%) presented symptoms. Jaundice was the most common finding (235; 26.9%), followed by anemia (10; 1.1%), and hepatomegaly (8; 0.9%). Sixteen newborns exhibited radiological abnormalities suggestive of CS-associated bone involvement (1.8%). However, most (607; 69.1%) were asymptomatic at birth and remained so during the hospital stay. 

 Almost all newborns received antibiotic treatment for CS (871; 99.1%) while hospitalized and were then discharged (862; 98.2%). The two most common antibiotic regimens used were 100,000 to 150,000 IU/kg/day of crystalline penicillin G for 10 days (669; 76.2%), and 50,000 IU/kg/day of procaine penicillin G for 10 days (116; 13.2%). In 13 cases (1.5%), a different antibiotic was used, such as ceftazidime, or ceftriaxone. Four cases (0.4%) resulted in fatalities attributable to syphilis during hospitalization, including one case of abortion and one case of stillbirth, which occurred due to extreme prematurity. 

## DISCUSSION

 The data presented herein reaffirm the persistent trend observed nationwide, with high rates of CS despite concerted public health efforts aimed at reducing the frequency of SiP. Notable among these efforts are the implementation of interventions in the context of maternal and child health programs and initiatives targeting sexually transmitted infections in primary health care efforts.^7,8^

 The present study found that the CS incidence at a single center ranged from 23.4 to 34.3 per 1,000 live births, significantly higher than the rate reported in Brazil in 2022 (10.3 cases per 1,000 live births).^8^ The data provides evidence of the current gap in achieving the target set by the World Health Organization, which aims to reduce worldwide CS incidence to 0.5 or fewer cases per 1,000 live births by 2030.^9^ It further emphasizes the need for additional measures to control the transmission of SiP amongst people in Salvador. 

 Regarding ethnicity, most mothers and newborns were reported as mixed-race or black, which is consistent with the ethnic profile of the city’s population. A recent study conducted in Brazil highlighted the disproportionate burden of maternal and congenital syphilis on non-white, low-income population segments.^10^ Similarly, recent observations in the United States have indicated a higher incidence of primary and secondary syphilis among black and Latino women compared to their white counterparts,^2^ highlighting the influence of social inequalities on infection epidemiology. 

 Notably, a significant proportion of cases (32.9%) occurred in mothers with complete or partial secondary level of education. An analysis of Brazilian Ministry of Health data on reported CS rates stratified according to education level reveals a significant growth in CS cases among children of mothers with secondary education between 2012 (32.8%) and 2022 (58.2%), with this demographic currently responsible for most CS cases in Brazil.^8^ A study by Soares and Aquino examined CS case reports from Bahia and demonstrated growth in the proportion of syphilis-positive mothers with secondary education from 27.7 to 33.5%, between 2014 and 2017, confirming that this same tendency is also occurring in the presently studied region.^11^

 Data on the time of diagnosis reveal that, despite an expansion of health services, intensified preventive measures, and awareness campaigns in recent years, diagnoses frequently occur late, as evidenced by the time of SiP diagnosis close to labor or postpartum in 38.4% of the mothers studied herein. Furthermore, the prevalence of inadequate treatment remains high (33.9%) in mothers and their sexual partners undergoing concurrent treatment. Brazilian Ministry of Health data support these findings, as SiP diagnosis has reportedly occurred around the time of labor or postpartum in 41.2% of cases between 2011–2022, while 51.3% of cases were inadequately treated.^8^ Of note, studies conducted in other states in the Northeast region of Brazil also emphasize the necessity for additional measures to urgently address the syphilis endemic.^12^

 In terms of laboratory diagnosis of CS in newborns, the frequency of positive non-treponemal tests in CSF was 4.3%, while radiological abnormalities suggestive of CS-associated bone involvement were noted in 4.0% of cases, close to the average rates cited in official reporting for the Northeast region (2.1% and 2.3%, respectively).^8^ Both findings have been associated with more severe CS outcomes in newborns. The frequency of symptomatic newborns was high (26.9%), with jaundice being the most common symptom observed. While jaundice can manifest due to various clinical conditions in newborns, it nonetheless warrants a thorough differential diagnosis. Importantly, cases of jaundice progressing to neonatal cholestasis and hepatosplenomegaly secondary to syphilis have been reported.^13^ Other studies have documented hepatosplenomegaly as a common symptom in newborns, underscoring the importance of investigating cases of jaundice associated with CS.^14^

 A significant limitation of this study is the incompleteness of compulsory notification records, which may reflect gaps between the time of medical diagnosis and the submission of notifications by hospital staff. Despite the inclusion of complementary data from patient electronic medical records, a high degree of incompleteness remained, particularly concerning sociodemographic and laboratory data for mothers and newborns. Of note, incomplete data regarding maternal treponemal test results were higher than that previously reported by Soares and Aquino for the Bahia state.^11^ Additionally, the data generated through mandatory reporting of CS cases does not include other pertinent clinical information, such as newborn anthropometric data, route of delivery, gestational age at delivery, congenital defects, etc., which could shed light on other deleterious effects associated with *T. pallidum* infection during pregnancy. 

 The study is also subject to selection bias, as data were obtained from a single referral maternity hospital. The high number of CS cases observed likely reflects, at least in part, the hospital’s role as a referral center for high-risk pregnancies, which concentrates on more complex cases, including those of CS. Consequently, while these findings highlight a significant burden of CS at this facility, they may not fully represent the true incidence of the disease in Salvador, as the rates could be overestimated due to the specialized patient profile in the hospital. Nonetheless, the persistently high number of cases underscores the ongoing public health challenge posed by CS in this population. 

 Although the study’s data describe a time before the SARSCoV-2 pandemic, the most recent epidemiological report by the Brazilian Ministry of Health^8^ showed that SiP and CS persist as a public health challenge for Brazil in the subsequent years. This study reinforces the importance of conducting broader investigations to assess the impact of CS in Bahia and throughout Brazil and highlights the need for intensified efforts regarding educational outreach to provide information about SiP and CS to pregnant women and their partners. It further emphasizes the necessity of enhancing access to high quality prenatal care, ensuring early, adequate diagnosis and treatment to limit the spread of the disease and eventually eliminate the transmission of syphilis from mother to child. 

## Data Availability

The database that originated the article is available with the corresponding author.
